# Dissociating breathlessness symptoms from mood in asthma

**DOI:** 10.1016/j.biopsycho.2021.108193

**Published:** 2021-10

**Authors:** Olivia K. Harrison, Lucy Marlow, Sarah L. Finnegan, Ben Ainsworth, Kyle T.S. Pattinson

**Affiliations:** a Translational Neuromodeling Unit, Institute for Biomedical Engineering, University of Zurich and ETH Zurich, Switzerland; b Department of Psychology, University of Otago, Dunedin, New Zealand; c Wellcome Centre for Integrative Neuroimaging, and Nuffield Division of Anaesthetics, Nuffield Department of Clinical Neurosciences, University of Oxford, United Kingdom; d Warwick Medical School, University of Warwick, Coventry, United Kingdom; e Department of Psychology, University of Bath, United Kingdom

**Keywords:** Asthma, Breathlessness, Mood, Interoception, Metacognition, Attention

## Abstract

It is poorly understood why asthma symptoms are often discordant with objective medical tests. Differences in interoception (perception of internal bodily processes) may help explain symptom discordance, which may be further influenced by mood and attention. We explored inter-relationships between interoception, mood and attention in 63 individuals with asthma and 30 controls. Questionnaires, a breathing-related interoception task, two attention tasks, and standard clinical assessments were performed. Questionnaires were analysed using exploratory factor analysis, and linear regression examined relationships between measures. K-means clustering also defined asthma subgroups. Two concordant asthma subgroups (symptoms related appropriately to pathophysiology, normal mood) and one discordant subgroup (moderate symptoms, minor pathophysiology, low mood) were found. In all participants, negative mood correlated with decreased interoceptive ability and faster reaction times in an attention task. Our findings suggest that interpreting bodily sensations relates to mood, and this effect may be heightened in subgroups of individuals with asthma.

## Highlights

• Symptoms and disease severity discordance is well recognised yet poorly understood.
• Interoception, is the way the brain perceives internal bodily sensations.
• Individual differences in interoceptive ability may help explain symptom discordance.
• In people with asthma, low mood was associated with decreased interoceptive ability.
• Our work indicates that interoception may be key to understanding breathlessness.

## 1 Introduction

Asthma is a common and often debilitating chronic condition that affects millions of people worldwide. Asthma is one of the most frequent chronic diseases, in particular amongst children, and has a global prevalence of approximately 1–18% (Braman, 2006). There is often a significant discrepancy between disease severity and the extent of symptom burden (Barnes et al., 2019, Bijl-Hofland et al., 1999, Boulay and Boulet, 2013, Chetta et al., 1998, De Peuter et al., 2005; in’t Veen et al., 1998; Salome et al., 2002; Steele, Meuret, Millard & Ritz, 2012; Teeter & Bleecker, 1998), and in up to 60% symptoms poorly reflect airway pathophysiology (Haldar et al., 2012; Janssens, Verleden, De Peuter, Van Diest & Van den Bergh, 2009). Moreover, symptom over- and under-perception does not appear to be a stable quality in the vast majority of individuals with asthma (Janssens et al., 2009). Therefore, in this study we wished to conduct a preliminary investigation into possible influential factors that may contribute to symptom discordance heterogeneity within asthma, potentially explaining any underlying variables that may be contributing to this inaccurate perception in relation to pathophysiology.

Affective dysfunctions such as increased anxiety and depression are some of the most significant co-morbidities in individuals with asthma (Agnihotri and Kant, 2019, Di Marco et al., 2011; Katon et al., 2007; Katon, Richardson, Lozano & McCauley, 2004; Rimington, Davies, Lowe & Pearson, 2001). It is thought that negative mood states (typical in anxiety and depression) may influence symptom perceptions through a variety of channels (Apter et al., 1997, Chen et al., 2006, De Peuter et al., 2007, De Peuter et al., 2007, Lavietes et al., 2000, Put et al., 2004, Rietveld and Everaerd, 2000, Rimington et al., 2001, Van den Bergh et al., 2004), including assigning wider (non-specific) symptoms to asthma diagnoses (Main, Moss-Morris, Booth, Kaptein & Kolbe, 2003), reduced perceptions of asthma control (Di Marco et al., 2010) and altered expectations regarding asthma symptoms (Brown et al., 2016, Marlow et al., 2019, Ritz and Steptoe, 2000).

**Fig. 1:**
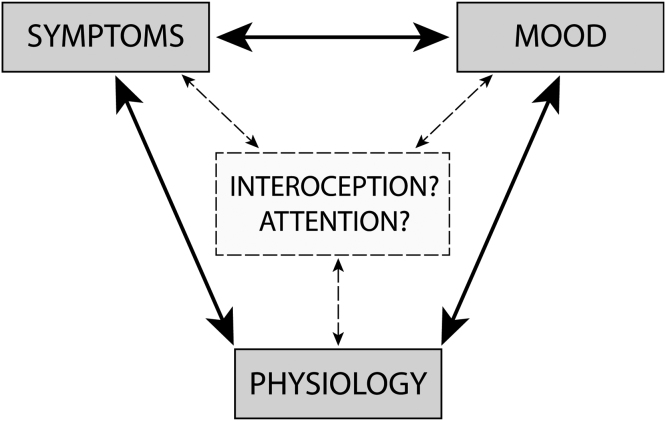
Visualisation of the potential place of interoception and attention within the interaction(s) between symptoms, mood and physiology.

Importantly, the interaction between physiological (dys)function and symptom perception depends critically on our ability to accurately sense, perceive and interpret afferent sensory information from our body, a process termed ‘interoception’ (Garfinkel et al., 2015, Khalsa et al., 2017, Seth, 2013). Therefore, the relationship between symptoms and physiology may be altered by our ability to accurately interpret these sensations, assign appropriate confidence to our judgements (metacognition), and/or by simply shifting our attention towards or away from them (Fig. 1) (Janssens et al., 2009). Furthermore, mood disorders themselves have been associated with differences in interoceptive abilities (Khalsa et al., 2017, Khalsa et al., 2018, Paulus, 2013, Paulus et al., 2019, Paulus and Stein, 2006) and attentional biases (Shechner et al., 2011) in the general population, while somatic symptom disorder has been associated with reduced interoceptive awareness, heightened attention towards symptoms and negative biases when interpreting bodily sensations (Paulus et al., 2019). Thus, mood may also alter interoception and/or attention towards sensations – either directly or indirectly through inflation of symptoms (Fig. 1) such as breathlessness. Furthermore, this relationship between symptoms, mood and physiology may differ across individuals, and any underlying systematic differences may give rise to symptom-based phenotypes of people with asthma.

Therefore, alongside standard clinical and physiological measures of asthma, here we assessed breathing-related interoceptive dimensions. These included a measure of sensitivity towards changes in inspiratory resistance, decision bias (towards over- or under-reporting the presence of a resistance), metacognitive bias (average confidence in decisions regarding the presence/absence of a resistance), and metacognitive sensitivity (correspondence between confidence ratings and performance accuracy) using the Filter Detection Task (Harrison et al., 2021) in combination with an established model of metacognition (Fleming, 2017). We additionally assessed the effect of both asthma-related fear words using the Visual Dot Probe Task (Cooper et al., 2011) and general spatial and temporal cues on attention using the Attention Network Task (Fan, McCandliss, Sommer, Raz & Posner, 2002)) and completed these assessments in both a group of individuals with asthma and healthy controls. General spatial and temporal models of attention identify three functional attentional networks: alerting (activating a vigilant state), orienting (directing cognitive resources towards salient stimuli) and executive control (higher level functions such as resolving conflicting stimuli), measured by the Attention Network Test (Fan et al., 2002). Additionally, the Visual Dot Probe Task (Cooper et al., 2011) examines allocation of attentional resources to affective (i.e. asthma-related) stimuli compared to neutral stimuli.

This study had the following aims:

• Aim 1, Interrelationships: To explore and assess the relationship between mood and symptom scores with physiological, interoceptive and attention measures in a cohort of individuals with asthma.
• Aim 2, Clustering: To assess whether sub-groups of individuals with asthma could be identified based on dissociable combinations of mood and symptom scores, denoting potential symptom-based ‘phenotypes’ within asthma.
• Aim 3, Separating mood and asthma: To investigate how mood is associated with breathing-related interoception and attention across all participants, and whether this relationship is altered with asthma (inner right triangle visualised in Fig. 1).

## 2 Methods

### 2.1 Participants

93 participants (58 female, mean age ± sd: Asthma = 44 ± 12 years, Controls = 44 ± 12 years, range 18–65 years) were recruited to the study through recruitment letters sent to patients with asthma from several GP practices and via poster advertisements. 63 participants had a doctor diagnosis of asthma; the remaining 30 participants were healthy with no significant disease or illness. Written informed consent was obtained from all participants prior to the start of the study. Study approval was granted by East Midlands – Nottingham 1 Research Ethics Committee (17/EM/0107 ID: 216046).

Study inclusion criteria: adults aged 18–65, healthy volunteers must not have asthma or a history of asthma. Participants with asthma must have active stable asthma and doctor diagnosis as reported by the participant. Exclusion criteria: Asthma exacerbation, significant cardiac, neurological, psychiatric or metabolic disease, other respiratory disease e.g. COPD or bronchiectasis, a smoking history of > 20 pack years, a history of prescription or non-prescription drug dependency, previous history of allergy or hypersensitivity to salbutamol or any of its components, history of cardiac tachyarrhythmia.

### 2.2 Data collection procedures

#### 2.2.1 Assessment schedule

Participants completed the following tasks included in this study:

1. Case report forms.
2. Questionnaire pack: State-Trait Anxiety Inventory (STAI) (Spielberger et al., 1970) (state then trait measures), Anxiety Sensitivity Index (ASI) (Reiss, Peterson, Gursky & McNally, 1986), The Center for Epidemiological Studies Depression Scale Revised (CESD-R-20) (Radloff, 1977), Health Anxiety Inventory (HAI) (Salkovskis, Rimes, Warwick & Clark, 2002), Multidimensional Assessment of Interoceptive Awareness (MAIA) (Mehling et al., 2012), Dyspnoea-12 (D-12) Questionnaire (Yorke, Moosavi, Shuldham & Jones, 2010), Nijmegen Questionnaire (NQ) (van Dixhoorn & Duivenvoorden, 1985), Fatigue Severity Scale (FSS) (Krupp, LaRocca, Muir-Nash & Steinberg, 1989). In addition to the above questionnaires, participants with asthma completed the following questionnaires: Catastrophic Thinking Scale in Asthma (CaA) (De Peuter, Lemaigre, Van Diest & Van den Bergh, 2008), Beliefs about Medicines Questionnaire (asthma) (BMQ) (Horne et al., 1997), Medication Adherence Scale (MAS) (Dolce et al., 1991, Morisky et al., 1986), Asthma Control Test (ACT) (Nathan et al., 2004) and Asthma Quality of Life Questionnaire (mini-AQLQ) (Juniper, Guyatt, Cox, Ferrie & King, 1999).
3. Attention tasks (Attention Network Task and Visual Dot Probe Task).
4. Filter detection task.
5. Bronchodilator reversibility test.
6. Exhaled nitric oxide test.
7. Blood sample for eosinophil levels.

#### 2.2.2 Physiological measures

Weight and height measurements were taken for all participants. Four millilitres of venous blood (whole blood) was acquired from the antecubital fossa by a trained researcher according to University of Oxford and Oxford University Hospitals venesection policy from both healthy volunteers and asthma group volunteers. The fraction of nitric oxide in exhaled breath (FeNO) was measured using a NIOX Mino device (Healthcare21, Basingstoke, UK). Complete spirometry assessment, including bronchodilator reversibility, was completed in all participants using a CareFusion micro spirometer (Cardinal Health, Chatham, Kent, UK). Spirometry measurements including Forced Expiratory Volume in 1 s (FEV1), Forced Vital Capacity (FVC) and peak flow were collected from participants before and after administration of a bronchodilator. In order to assess bronchodilator reversibility, following the first spirometry assessments, participants received 400 μg of salbutamol and the second session of spirometry measurements were taken 15 min following administration as per the European Respiratory Society guidelines (Pellegrino et al., 2005). At each spirometry assessment a minimum of three measurements in which the researcher confirmed correct technique were collected and the largest result was chosen for each measure (Miller et al., 2005). Participants were instructed to not use short-acting inhaled drugs for 4 h prior to the testing session and to not use long-acting β-agonist bronchodilators and oral therapy with aminophylline or slow-release β-agonists for 12 h prior to the testing session (Miller et al., 2005). A full medical history, including current medications, was taken from all participants.

#### 2.2.3 Questionnaires

All questionnaires were completed on paper print outs. All questionnaires were scored according to their respective manuals, descriptions of each questionnaire can be found in the Supplementary materials.

#### 2.2.4 Interoceptive Filter Detection Task

**Fig. 2:**
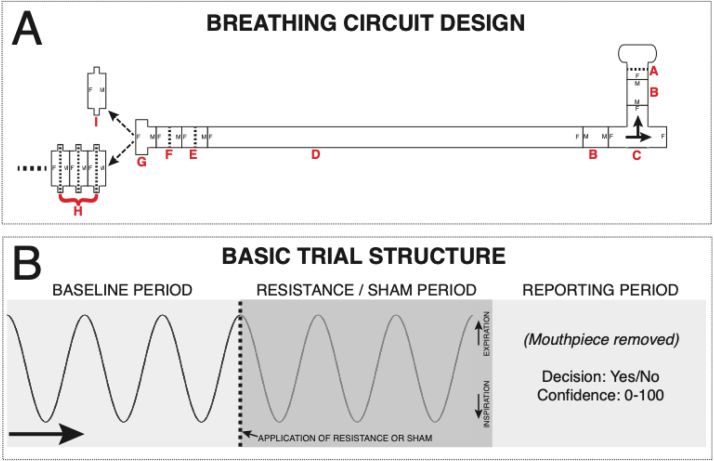
A) Diagram of circuitry for the filter detection task. A single-use, bacterial and viral mouthpiece (A) is attached to a 22 mm diameter connector (B) and a t-shaped inspiratory valve (C), connected to a 2 m length of 22 mm diameter flexible tubing (D) and two additional baseline filters (E and F). A 22–30 mm adapter (G) then allows the attachment of either a series of connected spirometry filters (H, Resistance at 30 L/min = 0.3 cm H_2_O) or a sham ‘dummy’ filter – a spirometry filter shell with the inner bacterial protection pad removed (I). B) Overview of the basic trial structure for a Yes/No formulation of the task. Participants take three normal size/pace breaths (with the sham filter attached), and during the third exhalation (indicated by the participant raising their hand and the dotted line in panel B) the experimenter either swaps the sham for a number of stacked filters (to provide a very small inspiratory resistance) or removes and replaces the sham filter. Following three more breaths, the participant removes the mouthpiece and reports whether they thought it a resistance was added (‘Yes’) or not (‘No’), and how confident they are in their decision on any scale (here 1–10 used, with 1 = guessing and 10 = maximally confident in their decision). If a two-interval forced choice (2IFC) formulation of the task is used, the filters (resistance) are either placed on the circuit for the first three breaths or the second three breaths according to the Filter Detection Task algorithm, with the sham filter on the system during the alternate period. The reported decision from the participant is whether they thought the resistance was on in either the first set or the second set of three breaths, and also again the confidence in their decision.

Participants completed an inspiratory respiratory resistance detection task based on the protocol used by Garfinkel and colleagues (Garfinkel et al., 2016), and outlined in further detail elsewhere (Harrison et al., 2021). Participants were asked to breathe through a breathing circuit (Fig. 2) and following a cue from the researcher determine if an inspiratory resistance was added, reporting their response and confidence in their decision. Inspiratory resistance was generated by the addition of spirometry filters (GVS, Lancashire, UK – Product 2800/17BAUF), following prior seminal work using inspiratory loading paradigms to measure sensitivity of breathing perception (Chou and Davenport, 2007, Daubenmier et al., 2013, Davenport et al., 2007, Garfinkel et al., 2016, Ruehland et al., 2019).

Figure panels A and B reproduced from Harrison et al., 2021 under Creative Commons licence.

The breathing system was set up as follows (in accordance with previously-outlined procedures (Harrison et al., 2021)): A single-use, bacterial and viral mouthpiece (PowerBreathe International Ltd., Warwickshire, UK – Product SKU PBF03) is attached to a 22 mm diameter connector (Intersurgical Ltd., Berkshire, UK – Product 1960000) and a t-shaped inspiratory valve (Hans Rudolf, Kansas City, MO, USA - Product 1410/112622), connected to a 2 m length of 22 mm diameter flexible tubing (Intersurgical Ltd. – Product 1573000) and two additional baseline filters (Intersurgical Ltd. – Product 1541000, and GVS, Lancashire, UK - Product 4222/03BAUA). A 22–30 mm adapter (Intersugical Ltd. – Product 197100) then allows the attachment of either a series of connected spirometry filters (GVS - Product 2800/17BAUF, pressure at 30 L/min = 0.3 cm H_2_O) or a sham ‘dummy’ filter – a spirometry filter shell with the inner bacterial protection pad removed. Further filters could be added to the system to increase resistance (minimum 1 filter and maximum 7 filters), or alternatively one filter with the mesh removed functioned as the dummy filter. Full details of the equipment used can be obtained from the Filter Detection Task (FDT) toolbox (https://github.com/ofaull/FDT) and from (Harrison et al., 2021).

On each trial within the task, participants were asked to take three normal breaths on the system at baseline where a single dummy filter was attached to the system. On their final exhalation at baseline, the participant would raise their hand to indicate that they had completed the baseline breaths. Once the participant raised their hand, the researcher either swapped the dummy with a replacement dummy or a number of real filters. Participants then took 3 further breaths on the system to determine if a resistance had been added or not. At the end of each trial (6 breaths in total), participants were asked to decide whether they thought a resistance had been added to the system (‘yes’) or not (‘no’). Participants were then further asked to specify their confidence in their decision on a scale of 0–100, where a complete guess was rated 0 and complete confidence was rated 100. Participants did not receive feedback on their accuracy/performance.

To complete the task, two or more practice trials were first completed following participant instructions. Task trials were then completed in blocks of 10. In each block, half the trials used a dummy filter on the test breaths and the other half used the test filters. For all participants, the first block of 10 trials compared a dummy filter to 4 resistance filters. To find a threshold for performance, the researcher aimed to find the filter level at which the participant performed at ~70% accuracy. Accuracy was calculated at the end of each block, if accuracy was below 60% correct, the number of filters used in the next block increased by one filter. If accuracy was above 80% correct, a resistance filter was removed for the next block. To complete the task, the aim was to complete 40–60 trials with a consistent number of resistance filters, where performance was held to between 60% and 80%.

#### 2.2.5 Attention tasks

**Fig. 3:**
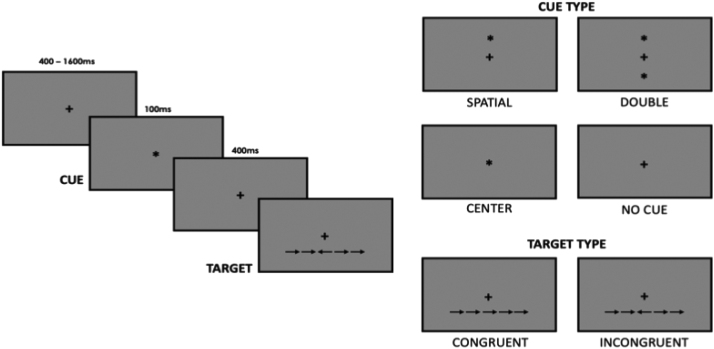
Visualisation of the Attention Network Task. A fixation cross is replaced by one of four cue conditions: a ‘centre cue’ (centre of the screen); a ‘double cue’ (two cues were presented above and below the fixation position); a ‘spatial cue’ (above or below the fixation position and was indicative of target stimulus location); or no cue (fixation cross remained). Following a 0.4 s pause, the target arrow plus two pairs of flanker arrows was presented above or below the fixation cross, with the flankers either congruent or incongruent with the target arrow. Participants were instructed to respond as quickly and as accurately as possible to the direction of the central arrow using right and left tabs on the keyboard.

**Fig. 4:**
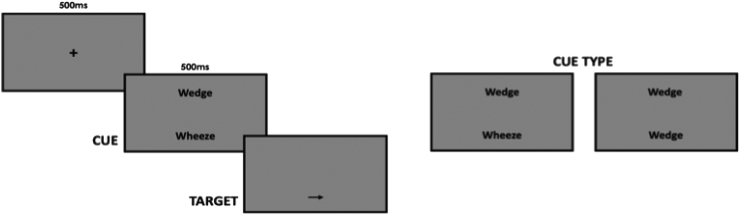
Visualisation of the Visual Dot Probe Task. A central fixation cross was presented for 0.5 s before being replaced by two words sitting above and below the fixation location. Either two neutral words or a neutral word and an asthma fear word were presented for 0.5 s, after which one word was replaced by a single arrow. Participants were required to respond as quickly and as accurately as possible to the direction of the arrow using the left and right arrow keys on the keyboard.

Participants completed two computer-based tasks to assess attention: The Attention Network Task (Fan et al., 2002) (Fig. 3) and the Visual Dot Probe Task (Cooper et al., 2011) (Fig. 4). Stimuli were presented using Inquisit 2 computer software and in both tasks, and participants responded to the direction of a target arrow by pressing the appropriate arrow key on the keyboard. Participants were asked to place their left index finger on the left arrow key and their right index finger on the right arrow key. Instructions for both tasks were presented visually on the screen prior to a practice session. Following each practice session, participants could clarify instructions and the researcher had opportunity to ensure understanding of the task.

In the Attention Network Task, participants viewed a central fixation cross for 400–1600 ms, followed by a cue (*) or no cue for 100 ms. Four cue conditions were used: a ‘centre cue’ where the cue replaced the fixation cross in the centre of the screen; a ‘double cue’ where two cues were presented above and below the fixation position; a ‘spatial cue’ where the cue was presented above or below the fixation position and was indicative of target stimulus location; or no cue was utilised. Following a 400 ms intermission (with the fixation cross still present), the target arrow plus two pairs of flankers were presented above or below the fixation cross. The flanker arrows were either congruent or incongruent with the direction of the target arrow. Participants were instructed to respond as quickly and as accurately as possible to the direction of the central arrow using right and left tabs on the keyboard.

The Visual Dot Probe Task utilised a set of asthma fear words and neutral words matched for word length and language frequency to assess attentional bias. A central fixation cross was presented for 500 ms before being replaced by two words sitting above and below the fixation location. Two neutral words, or a neutral word and an asthma fear word, were presented for 500 ms after which both words were removed with one being replaced by a single arrow stimulus. Participants were required to respond as quickly and as accurately as possible to the direction of the arrow using the left and right arrow keys on the keyboard.

### 2.3 Data analysis: calculation of summary measures

#### 2.3.1 Physiological measures

Predicted FEV1 and FVC values for each participant were calculated in line with Global Lung Initiative guidelines (Quanjer et al., 2012). Bronchodilator responsiveness was calculated as the percentage change in FEV1/FVC following administration of salbutamol (Barjaktarevic, Kaner, Buhr & Cooper, 2018). A full blood count analysis was conducted for the purpose of measuring blood eosinophils, which are a marker of asthma severity.

#### 2.3.2 Questionnaires

Questionnaires were first scored according to their respective manuals. A full correlation matrix was then calculated for (z-scored) questionnaires from the participants with asthma, using MATLAB 2017b (Mathworks, Natick, MA). The structure of the correlation matrices was first examined by applying a hierarchical cluster model to the data (Supplementary Fig. 1). Hierarchical models use the covariance across groups of measures in order to organize them spatially within the correlation matrix. The dataset was then visualized in Fig. 6 as a connectogram, containing a circular representation of interdependencies between measures. To formalize these relationships between questionnaire measures, an exploratory factor analysis was conducted, to uncover latent (hidden) factors. A quality assessment of the exploratory factor analysis model fit was performed by calculating the root mean square residual, Tucker-Lewis index and the root mean square error of approximation (Schreiber, Nora, Stage, Barlow & King, 2006), and comparing these metrics to established acceptable levels for these indices. Models were fit using Lavaan version 0.6-1 [E22] in R version 3.2.1 (R Core Team). See Supplementary material for further description of the exploratory factor analysis methods employed.

#### 2.3.3 Interoceptive Filter Detection Task

The Filter Detection Task was analysed using the hierarchical HMeta-d statistical model (Fleming, 2017), with model fits implemented in MATLAB (2017b) and sampling conducted using JAGS (Just Another Gibbs Sampler: v3.4.0). JAGS allows simulation and inversion of Bayesian hierarchical models using Markov Chain Monte Carlo (MCMC) sampling methods. To aid model fitting procedures, the confidence scores were down-sampled from 0 to 100 into 10-bin intervals. This model firstly utilizes signal detection theory (Green & Swets, 1966) to provide single subject parameter estimates for task difficulty (d′) and decision bias (*c*), where larger d′ indicates a greater discrimination between stimuli and a negative *c* indicates a bias towards reporting ‘yes’ (over-reporting the presence of a resistance), while a positive *c* denotes a bias towards reporting ‘no’ (under-reporting). Additionally, the model uses a hierarchical Bayesian formulation of metacognitive sensitivity, which is calculated by fitting the ‘metacognitive’ task difficulty parameter meta-d′, normalizing these values by single subject d′ to create estimates of Mratio (meta-d′/d′) that are independent of task performance, then taking the log_e_ of this metric. Metacognitive bias was also calculated as the average confidence score across the analysed trials.

#### 2.3.4 Attention tasks

**Fig. 5:**
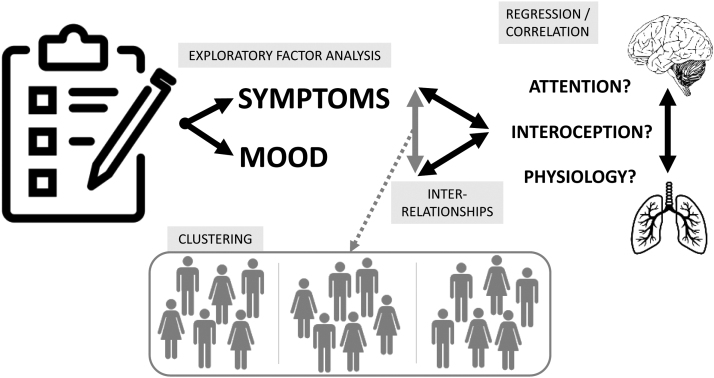
Infographic of study analysis procedures. Questionnaires were entered into an exploratory factor analysis, and two resulting factors that reflected ‘mood’ and ‘symptoms’ were identified. The relationship between each of these factors and measures of physiology, breathing-related interoception and attention were identified using correlation and regression analyses, addressing Study Aim 1 (interrelationships within asthma cohort). The relationship between symptoms and mood scores were then used to identify three subgroups within asthma using k-means clustering (Study Aim 2). Finally, regression analyses were used to separate the effects of mood and asthma in a combined cohort of individuals with asthma and healthy controls (Study Aim 3).

In the Attention Network Task, mean were calculated for each participant and each attentional condition (central, double, spatial and no cue). The alerting effect was calculated by subtracting the mean double cue reaction time from the mean no cue reaction time. The orienting effect was calculated by subtracting the mean spatial cue from the mean centre cue reaction time. The executive control effect was calculated by subtracting the mean congruent cue reaction time for each cue type from mean incongruent reaction time for each cue type. Incorrect trials and those with a reaction time lying beyond three standard deviations from the participant’s mean were excluded from analysis. Shorter times indicate quicker orienting, alerting and executive control skills. In the Visual Dot Probe Task, breathlessness interference scores were calculated by subtracting the mean response time for threatening asthma words from the reaction time in response to neutral asthma words for each participant (incorrect trials were removed from the analysis). Shorter times indicate quicker reactions induced by threatening asthma words. An overview of study analysis procedures is provided in Fig. 5 and further explanation of the measures is described in Table 1.

**Table 1:** Explanation of each of the measures calculated from the breathing-related interoception task and the two attention tasks.

Term	Explanation
*Filter detection task:*	
Number of filters (sensitivity)	The number of filters required for a participant to detect the presence of a resistance with 70% accuracy
Decision bias	A propensity to answer ‘yes’ (resistance present) or ‘no’ (no resistance present
Average confidence	Average confidence in perceptual decisions (i.e. metacognitive bias)
Metacognitive sensitivity (logMratio)	Correspondence between confidence scores and perceptual accuracy
	
*Attention network task:*	
Alerting score	The effect of the presence of an attention cue on changes in reaction time
Orienting score	The effect of a spatially-orienting cue on changes in reaction time
Executive score	The effect of incongruent cues (compared to congruent cues) on changes in reaction time
	
*Visual Dot Probe Task:*	
Breathlessness interference score	The effect of threatening asthma words compared to neutral asthma words on reaction time

### 2.4 Data analysis within asthma (Study Aims 1 and 2)

#### 2.4.1 Correlations between latent factors and physiology (Aim 1)

A correlation matrix was calculated between the factors identified from the questionnaire data and four physiological measures of FEV1/FVC, bronchodilator responsiveness (% change in FEV1/FVC), exhaled nitric oxide and blood eosinophils. Significance for each of the correlations was set at p < 0.05. As this study was exploratory, no corrections were applied for multiple comparisons.

#### 2.4.2 Asthma latent factor regression (Aim 1)

A set of linear regressions were then conducted to examine the relationship between the factors identified within the questionnaires and the held-out behavioural scores derived from the Filter Detection Task (filter number, decision bias, metacognitive bias and metacognitive sensitivity) and the attentional sub-domains (alerting, orienting, executive control and bias). The independent variables used in each of these analyses were the two latent factor scores from the exploratory factor analysis performed on the questionnaire data. For all except the metacognitive sensitivity analysis, regressions of the exploratory factor analysis scores for the latent factors were run against each of the behavioural scores using MATLAB’s fitlm function, with significance set at p < 0.05 and no corrections applied for multiple comparisons. As the metacognitive sensitivity scores are fit within a hierarchical model, we additionally performed an analogous hierarchical fit of a linear regression using the latent factor scores against interoceptive sensitivity (logMratio) (Harrison et al., 2021). Significance for these hierarchical regression coefficients were assessed using one-tailed 95% highest-density intervals on the regression (beta) parameters, to quantify any potential relationships between greater negative behavioural characteristics (such as breathing symptom scores or negative mood) and worsened metacognitive sensitivity (logMratio).

#### 2.4.3 Asthma sub-group stratification (Aim 2)

To investigate any possible stratification of participants with asthma based on the questionnaire scores, subject-wise clustering was performed on the exploratory factor analysis scores within the asthma group. The most statistically distinct groupings of participants were determined by Matlab’s evalcluster function, which utilises a k means clustering algorithm. Each of the groups were then compared to the control group for the physiological, interoceptive and attention measures using either independent *t*-tests or Wilcoxon rank sum tests (following tests for normal distributions of data), with significance taken at p < 0.05. A single logistic regression model was then applied to the asthma groups using MATLAB’s mnrfit function, with the following independent variables included in the model: peak flow, fraction of exhaled nitric oxide, blood eosinophil count, FEV1/FVC, FEV1%, bronchodilation, Attention Network Task alerting score, Attention Network Task orienting score, Attention Network Task executive score, Visual Dot Probe Task score, Filter Detection Task number of filters (sensitivity), Filter Detection Task decision bias, Filter Detection Task average confidence score, Filter Detection Task Mratio score. The group that scored the lowest on the symptom and mood factor scores was used as the pivot group for comparisons. Significance within the logistic regression was set at p < 0.05, and values are reported as FDR corrected and as exploratory uncorrected results.

Each asthma sub-group was additionally compared to the healthy volunteer group. For all measures except the Filter Detection Task metacognitive sensitivity, data was tested for normality using the Anderson-Darling test, with an alpha value of p < 0.05 used for rejecting the null hypothesis of normally distributed data. If the data were normally distributed the groups were compared using two-tailed independent *t*-tests, and if they were not normally distributed non-parametric Wilcoxon rank sum tests were employed. For metacognitive sensitivity scores, frequentist statistics cannot be employed as the values within each group were fit using separate hierarchical models. Therefore, to determine the significance of any group difference in these metacognitive sensitivity (logMratio) estimates, the highest-density intervals were calculated across the distribution of sample differences from each of the model fits (as previously described for the HMeta-d model (Fleming, 2017)). From this distribution of sample differences, a two-tailed 95% interval that does not span zero was used to determine any significant difference between the groups (Fleming, 2017). Significance was taken at p < 0.05, uncorrected for multiple comparisons.

### 2.5 Data analysis between asthma and healthy controls (Study Aim 3)

The effect of the latent variables identified were then investigated against the whole cohort of participants (asthma plus healthy controls). However, as the controls did not complete any asthma-specific questionnaires, only the latent mood factor was considered. As the controls were not fit within the exploratory factor analysis model, the first principal component (PC) of the questionnaire scores from the mood factor was calculated across asthma and control groups together (using only the questionnaires from the mood factor that were completed by both groups). A regression model was utilised that consisted of the following independent variables: a group difference regressor, the mood factor scores and an interaction Group*Mood regressor, which allows the simultaneous estimation of any difference between the groups, the effect of mood across all participants, and any difference in the effect of mood between the groups (interaction). We then ran this regression analyses on the held-out behavioural measures from the interoceptive and attention tasks, as described above. Significance of regression coefficients was taken at p < 0.05, uncorrected for multiple comparisons. For metacognitive sensitivity, the beta highest-density intervals were calculated across the distribution of samples for each of the three beta estimates in the model, and a two-tailed 95% highest-density interval that does not span zero was used to determine significance (Fleming, 2017). An additional simple group difference analysis between all individuals with asthma and all healthy controls using two-tailed independent *t*-tests or non-parametric Wilcoxon rank sum tests can be found in the Supplementary material.

### 2.6 Missing data

For all analyses except the between-group comparisons, missing data were imputed using the Markov chain Monte Carlo method (multiple imputation technique) within the MICE package in R (van Buuren & Groothuis-Oudshoorn, 2011). A summary of the percentage of missing data measures are provided in Supplementary Table 3.

## 3 Results

### 3.1 Study sample

The study population had well controlled mild to moderate asthma. A full description of the study sample can be found in Table 2, including age, BMI, physiological measures and questionnaire scores, as well as asthma-specific information.

**Table 2:** Clinical and questionnaire details for the asthma participants (*N* = 63) and healthy control participants (*N* = 30). Percentage of asthma or healthy control participants shown alongside absolute number. Variance is reported as mean ± standard deviation or median [interquartile range]. Asthma 'steps' as per definitions from the 2019 BTS/Sign guideline.

		Asthma		Healthy control
Age (years of age)		44 ± 12		44 ± 12
Gender (Female | Male)		39 | 24		19 | 11
BMI (kg/m^2^)		27 ± 6		24 ± 3
Peak Flow (L/min)		435 ± 125		406 ± 108
FeNO (ppb)		29 ± 20		24 ± 18
Eosinophil (× 10^9^ cells/litre)		0.13 ± 0.10		0.13 ± 0.12
FEV1% predicted		92 ± 26		101 ± 13
Asthma "step" (median [IQR])		2 [1]		
Number of participants "step 1"		13		
Number of participants "step 2"		25		
Number of participants "step 3"		22		
Number of participants "step 4"		3		
Number of participants "step 5"		0		
Alcohol (units per week):				
0		8 (13%)		4 (13%)
1–10		37 (59%)		17 (57%)
11–20		12 (19%)		9 (30%)
> 20		4 (6%)		–
Number of hours asleep daily				
< 6		14 (22%)		9 (30%)
> 6		48 (76%)		21 (70%)
Asthma specific demographic information				
Age of asthma diagnosis (years of age)				
0–10		29 (46%)		
11–20		11 (17%)		
> 20		23 (37%)		
Family history of asthma				
Yes		27 (43%)		
No		34 (54%)		
Number of asthma related adverse events				
1		2 (3%)		
Number of asthma related GP visits				
0		22 (35%)		
1–5		37 (59%)		
6–10		4 (6%)		
Number of asthma related hospital admissions				
1		14 (22%)		
Asthma triggers (number of participants in asthma group)				
Dust (40) (63%)	Exercise (39) (62%)	Pollen (34) (54%)		Smoke (28) (44%)
Pets (26) (41%)	Stress (24) (38%)	Food (11) (17%)		
Medication (number of participants in asthma group)				
Short-acting beta agonists (SABA) (59) (94%)			Long-acting beta agonists (LABA) (1) (2%)	
Inhaled steroids (28) (44%)			Oral steroids (2) (3%)	
Combined inhalers (steroid + LABA) (19) (30%)			Antimuscarinic inhaler (1) (2%)	
Leukotriene receptor antagonist (3) (5%)			Antihistamines (6) (10%)	
Antidepressants (6) (10%)			Diabetes medication (3) (5%)	
Cardiovascular medications (7) (11%)			Antibiotics (2) (3%)	
Antifungals (1) (2%)			Prescribed pain relief (1) (2%)	

### 3.2 Latent factors underlying questionnaire measures in asthma (Aim 1)

**Fig. 6:**
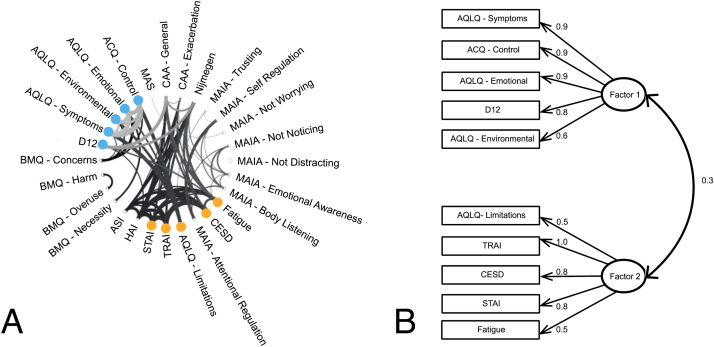
A) Connectogram of all questionnaire metrics collected in the participants with asthma (n = 63). Connections above R ≥ 0.35 are displayed, with line thickness denoting connection strength. The two significant latent factors are displayed using blue (Factor 1) and orange (Factor 2). B) Exploratory factor model structure, displaying the two latent factors with their questionnaire loadings and cross-loadings. Questionnaire abbreviations: D12, Dyspnoea 12; AQLQ, Asthma quality of life; ACQ, Asthma control questionnaire; MAS, Medicines adherence scale; CAA, catastrophising asthma; MAIA, Multidimensional assessment of interoceptive awareness; HAI, health anxiety inventory; ASI, anxiety sensitivity index; CESD, Centre for epidemiological depression questionnaire; TRAI, Trait anxiety inventory; STAI, State anxiety inventory; BMQ, Beliefs about medicines questionnaire.

An exploratory factor analysis performed on the questionnaire measures from the participants with asthma revealed the presence of two underlying latent factors (Fig. 6). One of these factors (Factor 1 in Fig. 6) consisted of scores of breathlessness symptoms (D12) and asthma quality of life measures (symptoms, control, environmental and emotional sub-scores), which we have summarised as an asthma ‘Symptom’ factor. The second factor included state/trait anxiety, depression, fatigue and the limitations sub-score from the asthma quality of life measures (Factor 2 in Fig. 6), which we have summarised as ‘Mood’ – although this factor is based largely on measures for habitual negative affect (excluding state anxiety). For both factors, increased factor scores represent elevated (worsened) measures of all loading questionnaires. All variables load strongly onto their factors and there is a small amount of correlation across the two factors. The fit statistics of this exploratory structural equation model are within acceptable bounds for the root mean square residual (RMSR < 0.08; here RMSR = 0.04), Tucker-Lewis Index (TLI > 0.9; here TLI = 0.95), while the root mean square error of approximation is marginal (RMSEA < 0.06; here RMSEA = 0.08). The root mean square error of approximation is an estimate of the discrepancy between the model and the data per degree of freedom for the model.

### 3.3 Relationship between mood/symptoms and physiology in asthma (Aim 1)

A correlation matrix between the two latent questionnaire factors and the six physiological measures within participants with asthma revealed only a significant correlation between symptom scores and blood eosinophils (R = 0.49, p < 0.001), with no physiological measures related to mood scores (Table 3). Mood and symptom scores were also moderately related (R = 0.32, p = 0.011), as previously reported in the exploratory factor model (Fig. 6). A full correlation matrix of all measured variables is provided in Supplementary Fig. 2.

**Table 3:** Correlation matrix in participants with asthma (Pearson’s correlation coefficient, R) between latent mood and symptom factors (from questionnaire data) and the physiological measures of FEV1/FVC, percentage predicted FEV1 (FEV1%), peak flow, bronchodilator responsiveness (BronchoR), fraction of exhaled nitric oxide (FeNO) and blood eosinophils.

	Mood	Symptoms	FEV1/FVC	FEV1%	Peak flow	BronchoR	FNO	Eosinophils
Mood		0.32*	0.11	-0.10	0.09	0.03	-0.16	-0.04
Symptoms			0.05	0.05	0.08	-0.10	-0.07	0.49*
FEV1/FVC				0.28*	0.03	-0.43*	-0.25*	-0.18
FEV1%					0.29*	-0.15	-0.22	-0.01
Peak flow						-0.11	0.08	-0.05
BronchoR							0.07	-0.16
FNO								0.10
Eosinophils								

* Denotes significance at *p* < 0.05.

### 3.4 Relationship between mood/symptoms and interoceptive and attention measures within asthma (Aim 1)

A set of exploratory analyses were then conducted where the two latent factors were regressed against all other behavioural measures collected in the FDT and attention tasks. None of these measures demonstrated a relationship with either of the factors, and the results from these analyses can be found in Table 4. As noted in the methods section, as the logMratio was fit with a hierarchical statistical model, a ‘significant’ result is denoted when the highest density interval does not span zero, signifying that 95% of the posterior distribution lies away from zero.

**Table 4:** Regression coefficients (betas) for the two-factor (mood and symptoms) regression models applied in the individuals with asthma. N.B. Metacognitive sensitivity (logMratio) was fit using a hierarchical regression model combined with a mcmc sampling procedure, and thus the highest density interval (HDI) is presented instead of a *p*-value. Significance was taken at *p* < 0.05 (or a 95% HDI that does not span zero) (two-tailed).

	‘Mood’ coefficient	T statistic	P value (or HDI)	Semi-partial R-squared
*Filter detection task:*				
Number of filters (sensitivity)	-0.16	-0.74	0.461	0.01
Decision bias	0.01	0.20	0.842	< 0.01
Average confidence	-3.46	-1.56	0.125	0.04
Metacognitive sensitivity (logMratio)	-0.28	NA	(− 0.64:0.05)	NA
*Attention network task:*				
Alerting score	-6.49	-1.05	0.299	0.02
Orienting score	-5.74	-0.86	0.394	0.01
Executive score	9.66	0.79	0.431	0.01
*Visual Dot Probe Task:*				
Breathlessness interference score	1.73	0.43	0.734	< 0.01
				
	‘Symptoms’ coefficient	T statistic	P value (or HDI)	Semi-partial R-squared
*Filter detection task:*				
Number of filters (sensitivity)	0.16	0.74	0.464	0.01
Decision bias	-0.02	-0.36	0.722	< 0.01
Average confidence	-1.73	-0.78	0.441	0.01
Metacognitive sensitivity (logMratio)	-0.08	NA	(− 0.38:0.19)	NA
*Attention network task:*				
Alerting score	-6.27	-1.01	0.315	0.01
Orienting score	-7.22	-1.08	0.284	0.02
Executive score	7.81	0.64	0.524	0.01
*Visual Dot Probe Task:*				
Breathlessness interference score	-3.01	-0.59	0.555	0.01

### 3.5 Sub-group stratification within asthma (Aim 2)

**Fig. 7:**
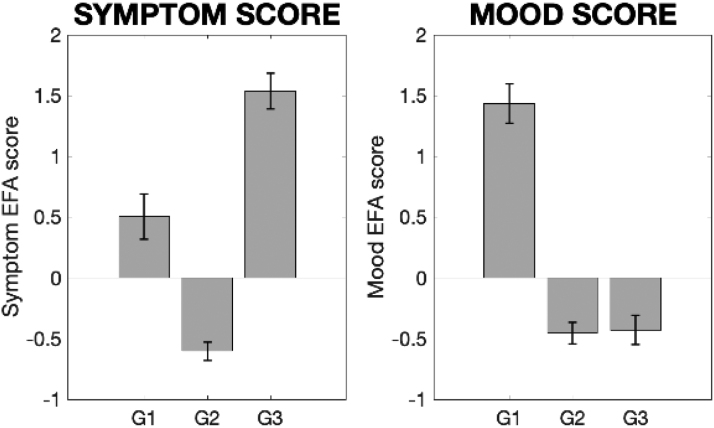
Asthma subgroup scores for the two latent factors identified in the exploratory factor analysis. Error bars denote standard error. Abbreviations: G1–G3, Groups 1–3.

Clustering individuals with asthma based on their latent factor scores revealed a three-group structure (Fig. 7 and Supplementary Fig. 3).

• Group 1 (discordant symptoms) demonstrated moderate symptom scores and high negative mood scores (i.e. more anxious/depressed etc) (Fig. 7), yet physiology measures equivalent to healthy controls.
• Group 2 (concordant symptoms) displayed the mildest symptom and mood scores.
• Group 3 (concordant symptoms) was characterised by the most severe symptom scores, high eosinophils, low bronchodilator reactivity yet low negative mood (i.e. less anxiety/depression etc).

**Fig. 8:**
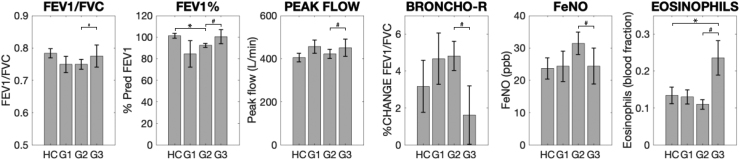
Group means and standard errors of the physiological measures for asthma subgroups (G1–G3) and healthy controls (HC). Each asthma sub-group was compared to healthy controls separately using independent *t*-tests or Wilcoxon rank sum tests, and the asthma sub-groups were compared using a logistic regression with Group 2 used as the pivot. N.B. Scores FEV1/FVC, percentage predicted FEV1 (FEV1%) and bronchodilator reversibility (broncho-r) were combined using a principal component analysis within the logistic regression due to high correlations. * Significantly different from control group using paired tests (p < 0.05). ^#^ Significantly different from Group 2 using a logistic regression within asthma groups (p < 0.05, FDR corrected for multiple comparisons). Abbreviations: FEV1%, percentage predicted FEV1; BRONCHO-R, bronchodilator responsiveness; FeNO, fraction of exhaled nitric oxide.

**Fig. 9:**
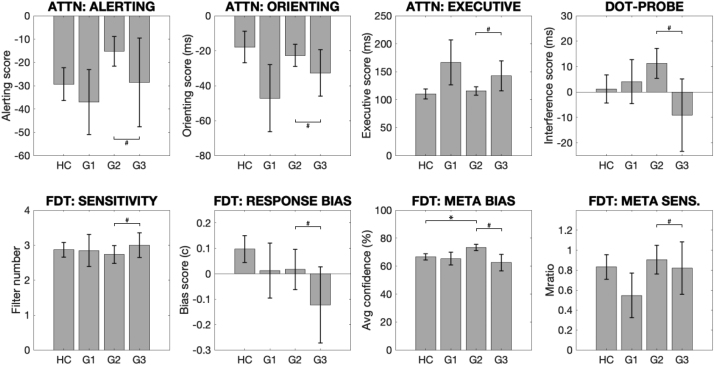
Group means and standard errors of the attention and interoceptive task measures for asthma subgroups (G1–G3) and healthy controls (HC). Each asthma sub-group was compared to healthy controls separately using independent *t*-tests or Wilcoxon rank sum tests, and the asthma sub-groups were compared using a logistic regression with Group 2 used as the pivot. N.B. Scores for metacognitive bias (Meta bias) and metacognitive sensitivity (Meta sens.) were combined using a principal component analysis within the logistic regression due to high correlations. * Significantly different from control group using paired tests (p < 0.05). ^#^ Significantly different from Group 2 using a logistic regression within asthma groups (p < 0.05, FDR corrected for multiple comparisons). Abbreviations: ATTN, attention task; VPT, Visual Dot Probe Task (Breathlessness interference score); FDT, filter detection task; META BIAS, metacognitive bias; META SENS., metacognitive sensitivity.
• Group 3 (10 participants; highest symptom scores and mild mood scores) was found to have elevated eosinophils yet similar spirometry measures (FEV1/FVC, predicted FEV1, bronchodilation and peak flow) (Fig. 8). Beyond the physiological measures, Group 3 also differed in all attention and interoceptive scores when compared to other asthma groups using a logistic regression, however no significant differences were found in these measures when compared to healthy controls (Fig. 9).

The sub-group scores were compared to healthy controls:

• Group 1 (15 participants; moderate symptoms, worst mood) did not demonstrate any significant differences when compared to healthy controls or Group 2 (using logistic regression).
• Group 2 (38 participants; mildest symptoms and mood scores, used as the pivot group in the logistic regression) demonstrated the typical decrease in predicted FEV1 compared to healthy controls (Fig. 8), and also showed increased metacognitive bias (average confidence) during the interoceptive task (Fig. 9).

A full table of the logistic regression results can be found in Supplementary Table 1. Additionally, group comparisons between healthy controls and the whole asthma cohort can be found in Supplementary Figs. 4–6.

Demographic characteristics of the asthma sub-groups were compared. There were no statistically significant differences between asthma sub-group means in age [F(2,60) = 1.2, p = 0.310], BMI [F(2,60) = 0.58, p = 0.565], or age of diagnosis [F(2,60) = 3.02, p = 0.056], as determined by one-way ANOVA’s. A Bartlett test revealed that the homogeneity of variances was violated for alcohol consumption (p = 0.000), number of hours asleep (p = 0.049). To account for this, Welch’s ANOVA’s were conducted - alcohol [F(2,60) = 2.0, p = 0.152] and number of hours asleep [F(2,60) = 0.78, p = 0.467]. A Welch’s ANOVA was also carried out for gender, which as a binary, categorical variable automatically violates assumptions of normality [F(2,60) = 0.32, p = 0.727]. A comparison of asthma related hospital admissions between the three asthma sub-groups was not statistically viable given the small numbers within each group. A summary of comparisons can be found in Supplementary Table 2.

### 3.6 Mood factor regression across asthma and healthy controls (Aim 3)

**Fig. 10:**
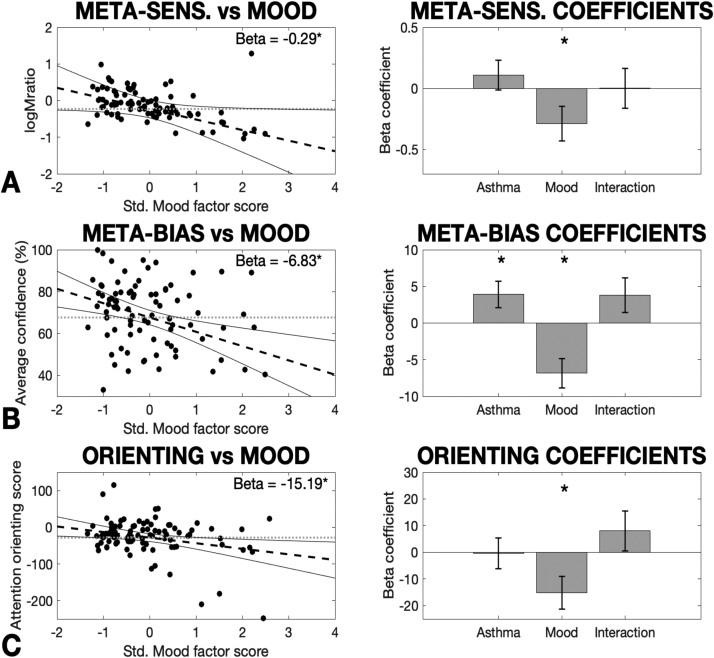
Significant results for the ‘Mood’ latent factor model regressed against the remaining measures: A) logMratio (representing metacognitive sensitivity), B) Average confidence (representing metacognitive bias), and C) Attention orienting. Additional regressors were included for any group difference between asthma and healthy controls, and also an interaction term between group and mood scores. In the left panels, dashed lines signify the regression line, dotted lines are plotted as a comparative ‘null model’ with an intercept term only (zero slope), and solid lines denote the 95% confidence interval of the regression line. The regression confidence intervals visually demonstrate the certainty regarding the estimate of the beta (slope) model parameter, where confidence intervals that do not encompass the ‘null model’ line are required for a significant effect. Error bars in the right panels denote standard error. Note: The logMratio regression was fit within a hierarchical model, which necessitates the use of the confidence intervals for a visual comparison of the uncertainty regarding model beta fits between the hierarchical and standard regression procedures. The average confidence and attention orienting regressions were fit using a standard linear regression model. In the right panels, the mean beta parameter estimate and standard error are shown. * Significantly different from zero (p < 0.05, two-tailed, uncorrected).

A set of regression analyses were then performed across the total cohort of participants, where each of the remaining behavioural measures were regressed against the mood factor scores, an asthma/control group factor, and an interaction between the two (all two-tailed tests). Firstly, a significant effect of the mood factor on logMratio (metacognitive sensitivity) was found across the total cohort of participants, while no effect of group nor any interaction effect was observed (Table 5; Fig. 10). Full regression results including regression coefficients, T statistics, p-values and semi-partial R^2^ metrics are reported in Table 5. This analysis also revealed a significant effect of the mood factor on average confidence (metacognitive bias) across the total cohort of participants, and accounting for mood also revealed a marginal group difference, where individuals with asthma reported higher confidence scores (Fig. 10). However, the interaction effect between mood and group did not reach significance (Table 5).

**Table 5:** Regression coefficients (betas) for the models (containing an asthma group difference regressor, a mood factor regressor and an interaction term) applied to the total cohort of individuals measured in this study (*n* = 93). N.B. Metacognitive sensitivity (logMratio) was fit using a hierarchical regression model combined with a mcmc sampling procedure, and thus the highest density interval (HDI) is presented instead of a *p*-value.

	Asthma coefficient	T statistic	P value (or HDI)	Partial R-squared
*Filter detection task:*				
Number of filters (sensitivity)	-0.05	-0.26	0.797	< 0.01
Decision bias	-0.02	-0.29	0.769	< 0.01
Average confidence	3.91	2.14	0.036*	0.05
Metacognitive sensitivity	0.11	NA	(− 0.12:0.35)	NA
*Attention network task:*				
Alerting score	0.63	0.13	0.901	< 0.01
Orienting score	-0.40	-0.07	0.944	< 0.01
Executive score	10.35	1.11	0.269	0.01
*Visual Dot Probe Task:*				
Breathlessness interference score	-0.69	-0.17	0.866	< 0.01
				
	‘Mood’ coefficient	T statistic	P value(or HDI)	Partial R-squared
*Filter detection task:*				
Number of filters (sensitivity)	0.03	0.15	0.882	< 0.01
Decision bias	-0.07	-1.18	0.242	0.02
Average confidence	-6.83	-3.43	0.001*	0.13
Metacognitive sensitivity	-0.29	NA	(− 0.57:− 0.02)*	NA
*Attention network task:*				
Alerting score	0.28	0.05	0.959	< 0.01
Orienting score	-15.19	-2.48	0.015*	0.06
Executive score	5.74	0.58	0.566	< 0.01
*Visual Dot Probe Task:*				
Breathlessness interference score	6.44	1.47	0.145	0.02
				
	Interaction coefficient	T statistic	P value (or HDI)	Partial R-squared
*Filter detection task:*				
Number of filters (sensitivity)	-0.07	-0.27	0.790	< 0.01
Decision bias	0.10	1.47	0.146	0.03
Average confidence	3.83	1.62	0.108	0.03
Metacognitive sensitivity	> 0.01	NA	(− 0.32:0.33)	NA
*Attention network task:*				
Alerting score	-13.03	-1.97	0.052	0.04
Orienting score	8.02	1.08	0.284	0.13
Executive score	8.40	0.69	0.491	0.01
*Visual Dot Probe Task:*				
Breathlessness interference score	-7.74	-1.45	0.151	0.02

* Significance was taken at *p* < 0.05 (or a 95% HDI that does not span zero (two-tailed)).

For the remaining measures, a significant effect of the mood factor was also found on the attention orienting score across the total cohort of participants, while there was no significant effect of group and the interaction effect did not reach significance (Table 5; Fig. 10). No other measures were found to be related to mood, group or an interaction of the two, with all regression results reported in Table 5.

## 4 Discussion

### 4.1 Main findings

In this study we firstly separated and characterised the degree of breathlessness symptoms and negative mood using self-report questionnaire measures, and assessed their relationship to measures of physiology, interoception and attention within asthma. Symptom scores were found to correlate with one physiological measure (blood eosinophils), while negative mood did not relate to any physiological measures. However, using these latent factor scores we revealed preliminary evidence for possible stratification of individuals with asthma into sub-groups, where these groups also demonstrated differences in both physiological, interoceptive and attention scores. Finally, negative mood was related to reduced interoceptive metacognitive sensitivity (or decreased ‘insight’ into breathing-related interoceptive abilities), decreased metacognitive bias (average confidence in interoceptive abilities) and attention orienting across all individuals (asthma and healthy controls), with only metacognitive bias elevated in individuals with asthma compared to healthy controls. These results may guide future studies and hypotheses regarding both the heterogeneity across individuals with asthma and research aimed at developing personalised treatments for breathlessness.

### 4.2 The relationship between symptoms, mood and asthma physiology

In asthma, the correspondence between the extent of physiological severity and self-report measures of symptom extent is known to be poor (Boulay & Boulet, 2013). Furthermore, there is a known association between asthma and elevated levels of anxiety and depression (Agnihotri and Kant, 2019, Di Marco et al., 2011; Katon et al., 2007, 2004; Rimington et al., 2001). However, here we revealed a clear dissociation of specific mood components (i.e. anxiety and depression) from symptom extent (reflected in asthma quality of life scores and breathlessness scores), with only a moderate correlation between these factors (Table 3). Consistent with much of the literature (Boulay & Boulet, 2013), symptom scores were only moderately related to one physiological measure of asthma severity (blood eosinophils), while mood scores were not related to any of the physiological measures. Physiology and self-report scores are only weakly related, and dissociating breathing symptoms from negative mood allows us to then investigate their independent relationships with important factors such as our ability to perceive bodily sensations (interoception), or our attention towards these perceptions. Importantly, it should be noted that this study was designed to assess how the broader aspects of physiology, symptoms and mood vary between individuals, rather than assessing the within-subject variance in these domains. However, as deficits in cognitive functions such as metacognition could cause both over- and under-perception, our findings between individuals might still be relevant for within-subject variability. This needs to be addressed with future work.

The variable relationship between symptom and negative mood factors also enabled the identification three sub-groups of individuals with asthma. Within these groupings, those who demonstrated the highest symptoms without a concurrent negative mood state were those individuals with the highest blood percentage of eosinophils, reduced bronchodilator responsiveness and more normalised resting spirometry and expired nitric oxide measures. Therefore, this group may represent those who are least responsive to typical inhaled bronchodilator medications, and thus have a greater level of physiological dysfunction during asthma exacerbations. While the results from these groupings are exploratory due to sample size, the differences between these sub-groups clearly demonstrates that the relationship between symptoms, mood and physiology is complex, and the variability between these measures is likely to contribute to the heterogeneity observed across the spectrum of asthma diagnoses. Furthermore, these results could underlie null effects seen in treatment trials with treatment programs suitable for one sub-group being masked by null effects in other individuals.

### 4.3 Interoception within the breathing domain

Interoception is an important gateway by which bodily sensations are connected to symptom perception, and here we investigated the relationship between asthma symptoms, mood and interoceptive domains. Within the breathing-related interoception task (Filter Detection Task), we firstly found that metacognitive bias (confidence) was related to both an asthma diagnosis and mood scores, while no direct relationships were observed between asthma symptoms and any interoceptive domains. Consistent with previous literature in exteroception (Rouault, Seow, Gillan & Fleming, 2018), a reduction in confidence regarding interoceptive decisions (metacognitive bias) was significantly related to negative mood across the cohort of both asthma and healthy controls. However, once this mood effect was accounted for, an *elevated* metacognitive bias (i.e. higher confidence scores) was observed within asthma, despite a more negative mood than healthy controls. While the direct interaction effect between metacognitive bias and the asthma group did not reach significance, these exploratory results indicate that there may be a difference in the confidence assigned to breathing perceptions within asthma. Interestingly, the difference in confidence was most pronounced in those individuals with asthma that reported the most positive mood and the least symptom scores (asthma Group 2). Therefore, it is possible that when exposure to elevated breathing symptoms in asthma is not coupled with worsened mood or self-report symptom burden, an elevation in perceptual confidence (metacognitive bias) can be induced even when absolute interoceptive sensitivity (i.e. the degree of inspiratory resistance that is able to be detected) does not change.

We additionally observed that negative mood was related to reduced metacognitive sensitivity in both asthma and healthy controls – a novel finding within the current metacognitive literature. Metacognitive sensitivity can be considered to reflect insight into one’s interoceptive performance, where an individual is able to more accurately assign greater confidence values on trials when they make correct judgements regarding interoceptive decisions (here the presence/absence of an inspiratory resistance), and lower confidence values when they make incorrect decisions. In previous work oriented towards the external domain (using a visual discrimination task), metacognitive sensitivity appeared to be unaffected by negative mood (Rouault et al., 2018) despite a decrease in metacognitive bias (overall confidence scores). In contrast, we have demonstrated both a reduction in metacognitive bias *and* sensitivity with negative mood scores within the interoceptive domain, indicating that the effect of negative mood may differentially alter external and internal metacognitive sensory processing. Furthermore, despite an overall more negative mood in asthma, the relationship between mood and metacognitive sensitivity is consistent between asthma and healthy controls, and thus may be a result of general mood factors such as anxiety and depression and independent of the presence of asthma.

Finally, while direct relationships were not identified between interoception and symptoms across the entire asthma cohort, the asthma sub-groups demonstrated important interoceptive differences. In particular, the asthma group with the highest symptoms and elevated blood eosinophils demonstrated a decrease in sensitivity towards detecting inspiratory resistances, a bias towards over-reporting the presence of a resistance, and a decrease in confidence (metacognitive bias) regarding these interoceptive decisions when compared to the low symptom asthma group. Therefore, while the relationship between interoception and symptoms may not be consistent across individuals, here we present evidence that breathing-related interoceptive properties may be disrupted in the presence of elevated asthma symptoms, although the causality of this relationship cannot be determined without a longitudinal intervention that targets these interoceptive abilities.

### 4.4 General and breathing-related attention

An additionally important aspect in our ability to perceive symptoms from our body is our capacity to attend to stimuli – both in general and in response to symptom-relevant stimuli. While we observed no relationship between either symptoms or mood and attention within asthma, across the total cohort of participants negative mood was associated with improved reaction times as a result of a spatial cue (measured using the attention ‘orienting’ score). Again, no group difference in the attention orienting score was apparent between asthma and healthy controls, indicating that this effect may also be associated with general changes in mood that are independent of asthma diagnosis. However, in a similar vein to the interoception results, the asthma group that displayed the greatest symptoms without a concurrent negative mood state (Group 3) exhibited differences in attention measures. Not only did these individuals have a greater effect of temporal and spatial cues on attention compared to the low symptom asthma group, they also demonstrated a greater bias towards asthma-related fear words in the Visual Dot Probe Task. While these results are exploratory in nature, they provide a platform for future work investigating the potential effect of worsened mood on increased attention towards spatial cues across the population, and also the possibility of altered attention in those who have elevated symptoms in asthma.

### 4.5 Further considerations and limitations

Our study population consisted largely of people with well-controlled mild-moderate asthma, in whom objective markers of airway inflammation were low. This suggests that our population mostly fell into non T-2 asthma phenotypes as identified in the literature. Although we are not able to perform more detailed phenotyping within the current dataset, our findings indicate that more detailed characterisation of mood and interoceptive factors would be extremely useful in future phenotyping efforts.

We note that Group 2 has higher FeNO than Group 3, whereas Group 3 has higher eosinophils than Group 2. This should be interpreted in the context of average values of both readings in both groups being below standard clinical thresholds, making it difficult to ascertain the importance of this observation. Only 10/63 participants recorded a FeNO above 40 parts per billion (a standard clinical threshold (NICE, 2017)), and in 5/63 were eosinophils above 0.3 × 10^9^ cells/litre (a level above which asthma attack rate becomes more common (Couillard, Jackson, Wechsler & Pavord, 2021)). Importantly, blood eosinophils may not fully represent airway eosinophil activity (Shrimanker et al., 2019), and a combined profile may prove more useful in predicting risk (Couillard et al., 2021, Shrimanker et al., 2019). With this in mind we have plotted FeNO against blood eosinophils in Supplementary Figure 7. Taking into consideration the guidance in Shrimanker et al. (2019), this suggests that only 3 of our sample are at considerable increased risk of asthma attack. We also note that in most parameters including lung function tests, none of the three asthma groups were different from healthy controls. Therefore, as our participant group were largely of mild-moderate severity and well controlled, we would not expect a difference.

The task employed to measure breathing-related interoception (the Filter Detection Task) is a newly-established protocol that is currently in under development (Harrison et al., 2021). One notable limitation within the current version of the task is the lack of physiological measures of respiratory flow and pressure, as the pressure differential generated across any static inspiratory resistance will be flow-dependent. Therefore, measures of interoceptive sensitivity (via the number of filters that were able to be detected) may be subject to the natural variations in breathing patterns across participants and between trials (Benchetrit, 2000, Bruce, 1996, Daubenspeck, 1981, Jaworski and Bates, 2019, Mador and Tobin, 1991). Therefore, measures of inspiratory pressure and flow could be recorded throughout this task, which would capture and allow us to quantify the changes in both the inspiratory pressure and flow (relative to the baseline breaths) that each participant utilised to detect the number of filters present. The use of mouth pressure could be used as a more accurate measure of interoceptive sensitivity, as inspiratory pressure will change in response to both the presence of a resistance as well as inter-participant and inter-trial inspiratory flow variability. However, it should also be noted that despite the lack of physiological measures, controlling the perceptual accuracy of each participant allows the metacognitive values to become independent of both the interoceptive sensitivity and breathing pattern employed. The properties of this task are discussed in further detail elsewhere (Harrison et al., 2021).

A further limitation of this study is the recruited sample size. Notably, the analysis technique of sub-group clustering is typically performed on sample sizes > 100 participants, and it is possible that the current results could be influenced by outliers in small sub-groups. Additionally, hierarchical regression techniques (such as was performed on the metacognitive Mratio score (Fleming, 2017)) require both moderate to large sample sizes and trial numbers to demonstrate significant effects (Harrison et al., 2021), and thus this study may be under-powered to identify small effects that may be present in the data.

## 5 Conclusions

A well-known discordance exists between symptom burden and objective measures of physiological dysfunction in asthma, with an elevated prevalence of co-morbidities such as anxiety and depression. Here we conducted preliminary tests to investigate whether both interoception and attention may be important mechanisms by which either symptoms or mood may alter the ability to accurately interpret sensory signals from the body. It appears that mood may directly influence aspects of both metacognition of interoception and general attention – important elements within perceptual pathways – in both asthma and healthy people. Lastly, we were able to utilise the variable relationship between symptoms and negative mood to identify sub-groups of individuals with asthma, who demonstrated distinct differences in physiological, interoceptive and attention measures. While small group sizes limit generalisability of these sub-groupings, we hope that these exploratory results may help generate hypotheses for future studies geared towards understanding the heterogeneity of symptom burden within asthma.

## Disclosure statement

Dr. Ben Ainsworth has received advisory board and honorarium with companies developing treatments for asthma: AstraZeneca. All remaining authors report no conflict of interests.
