# Supplementary material for: Dissociating breathlessness symptoms from mood in asthma
Source: Biol Psychol. 2021 Oct;165:None. doi: 10.1016/j.biopsycho.2021.108193 (PMC9355895; doi:10.1016/j.biopsycho.2021.108193)
Supplement: Supplementary file 1 — Supplementary material [file mmc1.docx]

*Supplementary Material:*

*Dissociating breathlessness symptoms from mood in asthma*

Olivia K. Harrison^1,2,3*^, Lucy Marlow^3,4*^, Sarah L. Finnegan^3^, Ben Ainsworth^5^, Kyle T. S. Pattinson^3^

^1^ Translational Neuromodeling Unit, Institute for Biomedical Engineering, University of Zurich and ETH Zurich, Switzerland

^2^ Department of Psychology, University of Otago, Dunedin, New Zealand

^3^ Wellcome Centre for Integrative Neuroimaging, and Nuffield Division of Anaesthetics, Nuffield Department of Clinical Neurosciences, University of Oxford, United Kingdom

^4^ Warwick Medical School, University of Warwick, Coventry, United Kingdom

^5^ Department of Psychology, University of Bath, United Kingdom

^*^ Authors contributed equally to the manuscript

**Supplementary Methods**

***Questionnaires***

**State-Trait Anxiety Inventory (STAI):** This is 2-part questionnaire with 20 items assessing trait anxiety and 20 items assessing state anxiety. Trait anxiety items are concerned with how the responder “generally” feels. State anxiety items are concerned with how the responder feels “right now” or “at this moment” (Spielberger et al., 1970).

**Anxiety Sensitivity Index (ASI):** This is a 16-item questionnaire which assesses the dispositional tendency to fear the symptoms of anxiety, viewing them as potentially harmful (anxiety sensitivity) (Reiss et al., 1986).

**The Center for Epidemiological Studies Depression Scale Revised (CESD-R-20):** This is a 20-item questionnaire which investigates depression (Radloff, 1977).

**Health Anxiety Inventory (HAI):** This is an 18-item questionnaire (short version) that assesses anxiety about health independently from health status (Salkovskis et al., 2002).

**Multidimensional Assessment of Interoceptive Awareness (MAIA):** This is a 32-item questionnaire that assesses self-report interoceptive awareness across 8 domains: Noticing, Not Distracting, Not Worrying, Attention Regulation, Emotional Awareness, Self-regulation, Body Listening, Trusting (Mehling et al., 2012).

**Dyspnoea-12 (D-12) Questionnaire:** This is a 12-item questionnaire which is designed to assess breathlessness severity. It has been validated in individuals with respiratory disease (Yorke et al., 2010).

**Nijmegen Questionnaire (NQ):** This questionnaire assesses dysfunctional breathing patterns associated with hyperventilation across 18 items (vanDixhoorn & Duivenvoorden, 1985).

**Fatigue Severity Scale (FSS):** This is a 9-item questionnaire that assesses participant fatigue which has been associated with respiratory disease (Krupp et al., 1989).

**Catastrophic Thinking Scale in Asthma (CaA):** This is a 13-item questionnaire adapted from the Pain Catastrophising Scale that assesses catastrophic thinking during an exacerbation (exacerbation scale) and in general (general scale) (DePeuter et al., 2008).

**Beliefs about Medicines Questionnaire (Asthma) (BMQ):** This is an 18-item questionnaire that assesses specific concerns and representations of medications prescribed to the individual (asthma specific) and general representations of medications (Horne et al., 1997).

**Medication Adherence Scale (MAS):** This is a 5-item questionnaire which assesses patient adherence to prescribed medication use for their asthma (Dolce et al., 1991; Morisky et al., 1986).

**Asthma Control Test (ACT):** This is a 5-item questionnaire that assesses how well the individual’s asthma is managed and controlled over a period of 4-weeks (Nathan et al., 2004).

**Asthma Quality of Life Questionnaire (mini-AQLQ):** This is a 15-item questionnaire that assesses the impact of asthma on an individual’s quality of life across 4 domains (Symptoms, Activities, Emotions and Environment) (Juniper et al., 1999).

***Cluster Analysis***

Hierarchical cluster models reorder variables based on their correlation strengths so that groups of related measures sit closer to each other than non-related measures. This allows natural relationships to be easily visualised. The modelling process formalises not only the relationship between pairs of variables, but also the manner by which shared variance can be described as part of larger, related clusters. The clustering algorithm initially considers pairs of variables in terms of their similarity or “distance” (in arbitrary units). Linked pairs are then incorporated into larger clusters with the goal of minimizing a cost function (distance to be bridged), a process that can be thought of as minimizing the dissimilarity within clusters. As pairs become clusters, a cluster tree or dendrogram is created. The distance between neighbouring branches indicates the relative similarity of two measures, while advancing up the hierarchical cluster tree moves further away in terms of link distance, and therefore similarity.

Hierarchical models are useful as a descriptive tool for examining and visualising the structure of the dataset as a whole. However, they do not provide information as to the significance of any given cluster of behavioural measures. In contrast, exploratory factor analysis, which falls under the umbrella of structural equation modelling, can be used to formalise the relationships observed in the hierarchical models. This allows the researcher to establish the presence of underlying shared constructs via a number of fit statistics without applying a preconceived structure on the result.

For the exploratory factor analysis, the smallest number of uncorrelated clusters that maximally explain the variance of the dataset was estimated. In this instance, parallel analysis with oblique rotation was employed to calculate this value. In a second step, the number of variables to be retained within the model was determined. A maximum likelihood estimation approach was applied, where variables that did not load significantly onto a particular factor or demonstrated significant cross loading (i.e. loaded onto more than one factor), were excluded from further testing. Finally, the model statistics were interrogated to formalize the shared variance across latent factors and the extent to which each variable contributed to its factor as a whole. The smallest number of factors that significantly explained the variance across the dataset was then accepted as the model of best fit. Model selection criteria included loading variables above 0.4 with no cross loading or freestanding variables, and significant X2/df ratio with Tucker-Lewis Index (TL-index) close to 1 and root mean square error of approximation < 0.06. Models were fit using Lavaan version 0.6-1 [E22] in R version 3.2.1 (R Core Team).

**Supplementary Results**

*Supplementary Figure 1. Clustergram of the questionnaire measures collected in the participants with asthma (n = 63). Questionnaire measures are ordered according to the strength of their correlation strength (R) so that any relationships between measures can be observed. Abbreviations: bmq, Beliefs about Medicines Questionnaire; maia, Multidimensional Assessment of Interoceptive Awareness; mas, Medication Adherence Scale; asi, Anxiety Sensitivity Index; caa; Catastrophic Thinking Scale in Asthma; fss, Fatigue Severity Scale; nijmegan, Nijmegen Questionnaire; hai, Health Anxiety Inventory; stai, State-Trait Anxiety Inventory (state anxiety subscale); trai, State-Trait Anxiety Inventory (trait anxiety subscale); cesd, The Center for Epidemiological Studies Depression Scale; acqlq, Asthma Quality of Life Questionnaire.*

*Supplementary Figure 2. Clustergram correlation matrix of all measured variables collected in the participants with asthma (n = 63). Measures are ordered according to the strength of their correlation strength (R) so that any relationships between measures can be observed. Abbreviations: bmq, Beliefs about Medicines Questionnaire; maia, Multidimensional Assessment of Interoceptive Awareness; mas, Medication Adherence Scale; asi, Anxiety Sensitivity Index; caa; Catastrophic Thinking Scale in Asthma; fss, Fatigue Severity Scale; nijmegan, Nijmegen Questionnaire; hai, Health Anxiety Inventory; stai, State-Trait Anxiety Inventory (state anxiety subscale); trai, State-Trait Anxiety Inventory (trait anxiety subscale); cesd, The Center for Epidemiological Studies Depression Scale; acqlq, Asthma Quality of Life Questionnaire; FDT, Filter Detection Task; executive, Attention task (executive subscale); orienting, Attention task (orienting subscale); alerting, Attention task (alerting subscale); asthmaBias, asthma bias measure from the Visual Dot Probe Task; FEV1/FVC, Forced expiratory volume in one second / Forced vital capacity; FEV1Pred, predicted FEV1; BronchoR, Bronchodilator reversibility; FeNO, Fraction of exhaled nitric oxide.*

*Supplementary Figure 3. Clustergram of the asthma subject stratification, based on the first principal components of the latent questionnaire factors identified using exploratory factor analysis (‘Mood’ and ‘Symptoms’), where three group clusters can be observed.*

*Supplementary Figure 4. Group means and standard errors for the first principal component score of the ‘Mood’ factor (identified in the exploratory factor analysis) between individuals with asthma and healthy controls. A larger score denotes higher measures on the contributing questionnaires and thus worsened mood. * Significantly different from control group (p < 0.05, uncorrected).*

*Supplementary Figure 5. Group means and standard errors of the physiological measures for individuals with asthma (AS) and healthy controls (HC). No scores were significantly different between groups (significance was taken at p < 0.05, uncorrected). Abbreviations: BRONCHO-R, bronchodilator responsiveness; FeNO, fraction of exhaled nitric oxide. Eosinophils blood fraction represents number of cells *10^9^ per litre.*


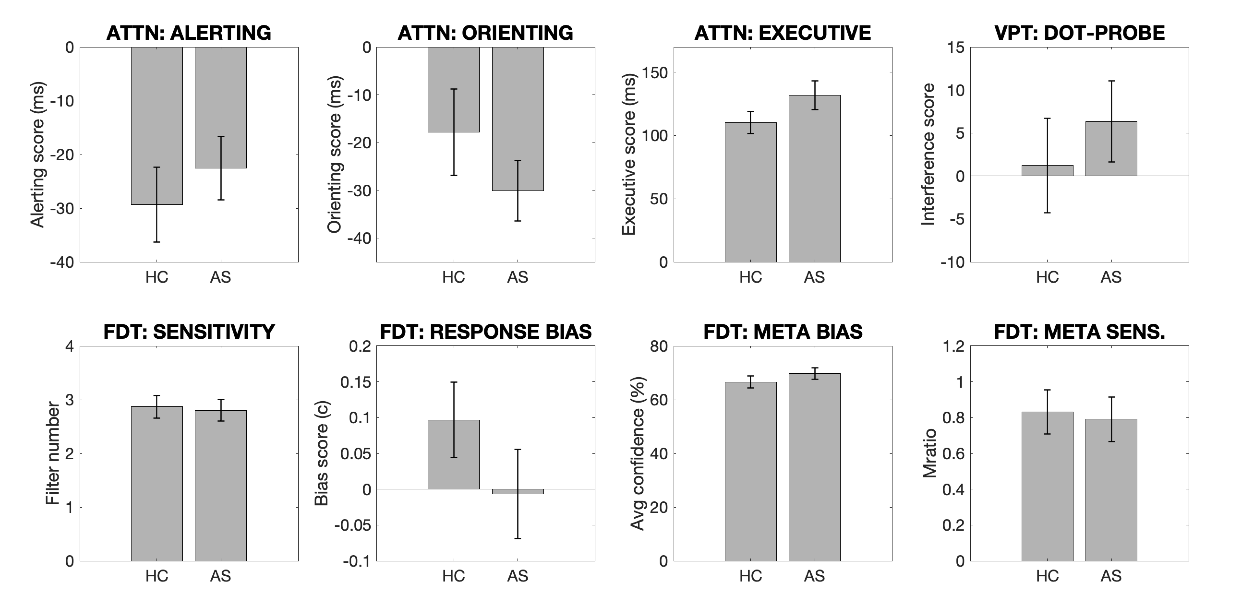


*Supplementary Figure 6. Group means and standard errors of the attention and interoceptive task measures for individuals with asthma (AS) and healthy controls (HC). No scores were significantly different between groups (significance was taken at p < 0.05, uncorrected). Abbreviations: ATTN, attention task; VPT,* *Visual Dot Probe Task; FDT, filter detection task; META BIAS, metacognitive bias; META SENS., metacognitive sensitivity.*


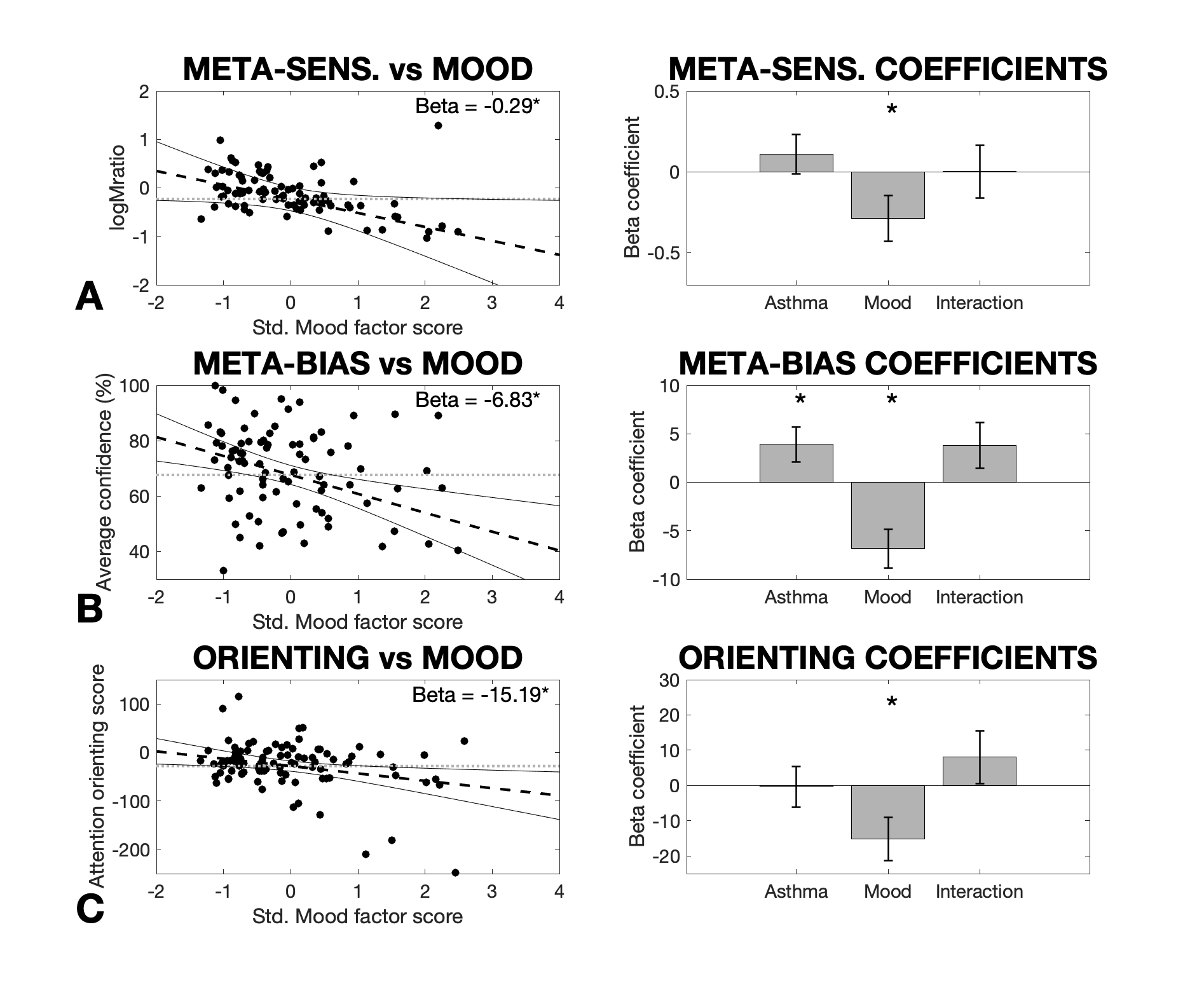


*Supplementary Figure 7. Significant results for the ‘Mood’ latent factor model regressed against the remaining measures: A) logMratio (representing metacognitive sensitivity), B) Average confidence (representing metacognitive bias), and C) Attention orienting. Additional regressors were included for any group difference between asthma and healthy controls, and also an interaction term between group and mood scores. In the left panels, dashed lines signify the regression line, dotted lines are plotted as a comparative ‘null model’ with an intercept term only (zero slope), and solid lines denote the 95% confidence interval of the regression line. The regression confidence intervals visually demonstrate the certainty regarding the estimate of the beta (slope) model parameter, where confidence intervals that do not encompass the ‘null model’ line are required for a significant effect. Error bars in the right panels denote standard error. Note: The logMratio regression was fit within a hierarchical model, which necessitates the use of the confidence intervals for a visual comparison of the uncertainty regarding model beta fits between the hierarchical and standard regression procedures. The average confidence and attention orienting regressions were fit using a standard linear regression model. In the right panels, the mean beta parameter estimate and standard error are shown. * Significantly different from zero (p < 0.05, two-tailed, uncorrected).*

*Supplementary Table 1. Logistic regression coefficients for asthma sub-group comparisons, where each of groups 1 and 3 were compared to group 2 (lowest mood and symptom scores). * Significant under the exploratory threshold of p < 0.05, and* ***^#^*** *Significant with FDR-correction for multiple comparison applied.*

|  | G1 v. G2  coefficient | P value | G3 v. G2  coefficient | P value |
| --- | --- | --- | --- | --- |
| Intercept | -1.46 | <0.01 | -306.63 | <0.001^#^ |
| Physiological measures |  |  |  |  |
| Peak flow | 0.69 | 0.19 | -3.46 | <0.001^#^ |
| Fraction exhaled nitric oxide | 0.94 | 0.13 | 74.64 | <0.001^#^ |
| Blood eosinophils | 0.84 | 0.30 | -138.18 | <0.001^#^ |
| PCA1 *(FEV1/FVC + FEV1% + Bronchodilation)* | -0.57 | 0.14 | 147.70 | <0.001^#^ |
| Attention network task |  |  |  |  |
| Alerting score | -0.81 | 0.06 | 44.29 | <0.001^#^ |
| Orienting score | -0.51 | 0.31 | -22.46 | <0.001^#^ |
| Executive score | -1.13 | 0.11 | 11.05 | <0.001^#^ |
| Visual Dot Probe Task |  |  |  |  |
| Breathlessness interference score | 0.57 | 0.27 | 93.99 | <0.001^#^ |
| Filter detection task |  |  |  |  |
| Number of filters (sensitivity) | -0.29 | 0.49 | 2.95 | <0.001^#^ |
| Decision bias | -0.49 | 0.37 | -81.44 | <0.001^#^ |
| PCA2 *(Confidence & Mratio)* | -0.10 | 0.79 | -102.27 | <0.001^#^ |

*Supplementary Table 2. General demographic information for healthy controls and asthma group including sub-groups. There were no statistically significant differences between asthma sub-group means in age, BMI, peak flow, FeNO and age of diagnosis. Full statistical analysis of sub-group comparisons is included in the main text.*

|  | Asthma | Healthy control |
| --- | --- | --- |
| Number of participants | Total: 63 | Total: 30 |
|  | Group 1: 15 |  |
|  | Group 2: 10 |  |
|  | Group 3: 38 |  |
| Age (years) | Total: 44 ± 12 | Total: 44 ± 12 |
|  | Group 1: 40 ± 10 |  |
|  | Group 2: 48 ± 16 |  |
|  | Group3: 44 ± 12 |  |
| Gender (Female \| Male) | Total: 39 \| 24 | Total: 19 \| 11 |
|  | Group 1: 10 \| 5 |  |
|  | Group 2: 7 \| 3 |  |
|  | Group 3: 22 \| 16 |  |
| BMI (kg/m^2) | Total: 27 ± 6 | Total: 24 ± 3 |
|  | Group 1: 29 ± 9 |  |
|  | Group 2: 26 ± 7 |  |
|  | Group3: 27 ± 6 |  |
| Alcohol (units per week) | Total: 11 ± 28 | Total: 7 ± 6 |
|  | Group 1: 5 ± 6 |  |
|  | Group 2: 8 ± 7 |  |
|  | Group 3: 14 ± 35 |  |
| Number of hours asleep (daily) | Total: 7 ± 1 | Total: 7 ± 2 |
|  | Group 1: 7 ± 1 |  |
|  | Group 2: 8 ± 1 |  |
|  | Group 3: 7 ± 2 |  |
| Asthma hospital admisions (pa) | Total: 0.03 ± 0.2 | N/A |
|  | Group 1: 0.1 ± 0.3 |  |
|  | Group 2: 0.1 ± 0.3 |  |
|  | Group 3: 0 ± 0 |  |
| Age of diagnosis (years) | Total: 19 ± 16 | N/A |
|  | Group 1: 21 ± 17 |  |
|  | Group 2: 29 ± 20 |  |
|  | Group 3: 15 ± 15 |  |
| FEV1 % predicted | Total: 92 ± 26 | Total: 101 ± 13 |
|  | Group 1: 84.5 ± 47 |  |
|  | Group 2: 101 ± 20 |  |
|  | Group3: 92 ± 12 |  |
| Peak Flow (L/min) | Total: 435 ± 125 | Total: 406 ± 108 |
|  | Group 1: 456 ± 118 |  |
|  | Group 2: 451 ± 124 |  |
|  | Group3: 422 ± 131 |  |
| FeNo (ppb) | Total: 29 ± 20 | Total: 24 ± 18 |
|  | Group 1: 24 ± 18 |  |
|  | Group 2: 24 ± 18 |  |
|  | Group3: 32 ± 21 |  |
| Eosinophil (cells x10*9/L) | Total: 0.13 ± 0.10 | Total: 0.13 ± 0.12 |
|  | Group 1: 0.13 ± 0.10 |  |
|  | Group 2: 0.20 ± 0.15 |  |
|  | Group3: 0.11 ± 0.10 |  |
| Asthma 'step' | Total 2 [1] |  |
|  | Group 1: 2[1] |  |
|  | Group 2: 3[0.75] |  |
|  | Group 3: 2[2] |  |
| Number of participants in each step | Total: 13,25,22,3,0 |  |
| (step 1, step 2, step 3, step 4, step 5) | Group 1: 1,7,7,0,0, |  |
|  | Group 2: 1,2,5,2,0 |  |
|  | Group 3: 11,16,10,1,0 |  |

*Supplementary Table 3. Missing data (reported as a percentage of data points) for each of the measures collected in both asthma and healthy controls.*

|  | Asthma | Healthy controls |
| --- | --- | --- |
| Questionnaires | 0.3 | 1.1 |
| FEV1/FVC | 0 | 0 |
| FEV1 % predicted | 1.6 | 0 |
| Peak flow | 1.6 | 3.3 |
| Bronchodilator responsiveness | 0 | 0 |
| FeNO | 4.8 | 10 |
| Eosinophils | 11.1 | 10 |
| Attention task | 0 | 0 |
| Visual probe task | 0 | 0 |
| Filter detection task | 4.8 | 0 |

Supplementary Figure 7. Relationship between blood eosinophils and FeNO in the study sample. High values in both markers have a stronger predictive value for asthma attacks (Shrimanker et al., 2019)

**References**

De Peuter, S., Lemaigre, V., van Diest, I. & van den Bergh, O. (2008). Illness-specific catastrophic thinking and overperception in asthma. *Health Psychology*, *27*(1), 93–99. https://doi.org/10.1037/0278-6133.27.1.93

Dolce, J. J., Crisp, C., Manzella, B., Richards, J. M., Hardin, J. M. & Bailey, W. C. (1991). Medication Adherence Patterns in Chronic Obstructive Pulmonary Disease. *Chest*, *99*(4), 837–841. https://doi.org/10.1378/chest.99.4.837

Horne, R., Weinman, J. & Hankins, M. (1997). The beliefs about medicines questionnaire: The development and evaluation of a new method for assessing the cognitive representation of medication. *Psychology and Health*, *14*(1), 1–24. https://doi.org/10.1080/08870449908407311

Juniper, E. F., Guyatt, G. H., Cox, F. M., Ferrie, P. J. & King, D. R. (1999). Development and validation of the Mini Asthma Quality of Life Questionnaire. *European Respiratory Journal*, *14*(1), 32–38. https://doi.org/10.1034/j.1399-3003.1999.14a08.x

Krupp, L. B., LaRocca, N. G., Muir-Nash, J. & Steinberg, A. D. (1989). The Fatigue Severity Scale: Application to Patients With Multiple Sclerosis and Systemic Lupus Erythematosus. *Archives of Neurology*, *46*(10), 1121–1123. https://doi.org/10.1001/archneur.1989.00520460115022

Mehling, W. E., Price, C., Daubenmier, J. J., Acree, M., Bartmess, E. & Stewart, A. (2012). The Multidimensional Assessment of Interoceptive Awareness (MAIA). *PLOS ONE*, *7*(11), e48230-22. https://doi.org/10.1371/journal.pone.0048230

Morisky, D. E., Green, L. W. & Levine, D. M. (1986). Concurrent and predictive validity of a self-reported measure of medication adherence. *Medical Care*, *24*(1), 67–74.

Nathan, R. A., Sorkness, C. A., Kosinski, M., Schatz, M., Li, J. T., Marcus, P., Murray, J. J. & Pendergraft, T. B. (2004). Development of the asthma control test: A survey for assessing asthma control. *Journal of Allergy and Clinical Immunology*, *113*(1), 59–65. https://doi.org/10.1016/j.jaci.2003.09.008

Radloff, L. S. (1977). The CES-D scale a self-report depression scale for research in the general population. *Applied Psychological Measurement*, *1*(3), 385–401.

Reiss, S., Peterson, R. A., Gursky, D. M. & McNally, R. J. (1986). Anxiety sensitivity, anxiety frequency and the prediction of fearfulness. *Behaviour Research and Therapy*, *24*(1), 1–8. https://doi.org/10.1016/0005-7967(86)90143-9

Salkovskis, P. M., Rimes, K. A., Warwick, H. M. C. & Clark, D. M. (2002). The Health Anxiety Inventory: development and validation of scales for the measurement of health anxiety and hypochondriasis. *Psychological Medicine*, *32*(5), 843–853. https://doi.org/10.1017/s0033291702005822

Shrimanker, R., Keene, O., Hynes, G., Wenzel, S., Yancey, S. & Pavord, I. D. (2019). Prognostic and Predictive Value of Blood Eosinophil Count, Fractional Exhaled Nitric Oxide, and Their Combination in Severe Asthma: A Post Hoc Analysis. *American Journal of Respiratory and Critical Care Medicine*, *200*(10), 1308–1312. https://doi.org/10.1164/rccm.201903-0599le

Spielberger, C. D., Gorsuch, R. L. & Lushene, R. E. (1970). *State-trait anxiety (STAI) manual*. Palo Alto.

van Dixhoorn, J. & Duivenvoorden, H. J. (1985). Efficacy of Nijmegen questionnaire in recognition of the hyperventilation syndrome. *Journal of Psychosomatic Research*, *29*(2), 199–206. https://doi.org/10.1016/0022-3999(85)90042-x

Yorke, J., Moosavi, S. H., Shuldham, C. & Jones, P. W. (2010). Quantification of dyspnoea using descriptors: development and initial testing of the Dyspnoea-12. *Thorax*, *65*(1), 21–26. https://doi.org/10.1136/thx.2009.118521
